# Ethical Considerations for Equitable Access to Genomic Sequencing for Critically Ill Neonates in the United States

**DOI:** 10.3390/ijns8010022

**Published:** 2022-03-21

**Authors:** Kristen P. Fishler, Joshua C. Euteneuer, Luca Brunelli

**Affiliations:** 1 Munroe-Meyer Institute for Genetics and Rehabilitation, University of Nebraska Medical Center, Omaha, NE 68198, USA; 2 Methodist Women’s Hospital, Omaha, NE 68022, USA; joshua.euteneuer@nmhs.org; 3 Division of Neonatology, University of Utah School of Medicine, Salt Lake City, UT 84132, USA; luca.brunelli@hsc.utah.edu

**Keywords:** ethics, diagnostic odyssey, newborn genomic sequencing, NICU, health disparities, equity, justice

## Abstract

Rare diseases impact all socio-economic, geographic, and racial groups indiscriminately. Newborn screening (NBS) is an exemplary international public health initiative that identifies infants with rare conditions early in life to reduce morbidity and mortality. NBS theoretically promotes equity through universal access, regardless of financial ability. There is however heterogeneity in access to newborn screening and conditions that are screened throughout the world. In the United States and some other developed countries, NBS is provided to all babies, subsidized by the local or federal government. Although NBS is an equitable test, infants admitted to neonatal intensive care units (NICUs) may not receive similar benefits to healthier infants. Newborns in the NICU may receive delayed and/or multiple newborn screens due to known limitations in interpreting the results with prematurity, total parenteral nutrition, blood transfusions, infection, and life support. Thus, genomic technologies might be needed in addition to NBS for equitable care of this vulnerable population. Whole exome (WES) and genome sequencing (WGS) have been recently studied in critically ill newborns across the world and have shown promising results in shortening diagnostic odysseys and providing clinical utility. However, in certain circumstances several barriers might limit access to these tests. Here, we discuss some of the existing barriers to genomic sequencing in NICUs in the United States, explore the ethical implications related to low access, consider ways to increase access to genomic testing, and offer some suggestions for future research in these areas.

## 1. Introduction

In the United States and across the globe, newborn screening (NBS) is a successful public health program to identify infants with rare genetic conditions early in life with the goal of reducing morbidity and mortality. In this way, newborn screening programs promote equity and protect vulnerable infants, regardless of the financial abilities of the family. However, there is heterogeneity in access to newborn screening as well as the conditions that are screened throughout the world [1]. Some developing countries have yet to initiate a newborn screening program due to limited infrastructure to conduct research and/or financial capability to fund treatments, widening global equity gaps [2]. In the United States and some other developed countries, NBS is provided to all babies, subsidized by the local or federal government. Rare diseases impact all socio-economic, geographic, and racial groups indiscriminately, with NBS providing an opportunity to promote equity within and across these groups.

The United States is one of the countries in which the newborn screening program is supported through national recommendations for conditions which should be screened [1]. Although NBS is an equitable test in healthy newborns, there are instances in the United States in which some infants do not receive the same benefit. For example, infants admitted to a neonatal intensive care unit (NICU) shortly after birth may receive delayed results and/or require multiple newborn screens due to known limitations in interpreting the results in infants who are premature, receive total parenteral nutrition, have a current infection, have received a blood transfusion, require life support, etc. [3]. Thus, the most vulnerable infants with higher risks of testing positive on newborn screening may be disadvantaged compared to healthy infants. In addition, the limited testing on NBS may not be sufficient to diagnose the cause of disease in critically ill infants with multiple congenital anomalies and/or other causes of morbidity and mortality. These considerations suggest that approaches such as genomic technologies might be needed in the vulnerable NICU population in addition to NBS.

Whole exome (WES) and genome sequencing (WGS) have been recently studied in critically ill newborns in NICUs across the world, with investigations into the clinical utility, cost effectiveness, diagnostic utility, etc. More than 20 clinical trials have been performed, with more currently planned [4]. In critically ill newborns, genomic sequencing has well established diagnostic yield and potential clinical utility [5,6,7,8,9]. In the NICU, the diagnostic yield of these next-generation sequencing tests is between 21% and 58% [9,10,11,12,13,14,15,16,17]. Changes to medical management have been reported in 28–32% of infants with diagnoses [9,17,18]. Other studies have suggested that genomic sequencing might reduce downstream healthcare costs by facilitating targeted and preventative medical care and avoiding costly, unnecessary procedures [18,19,20,21]. As a result of these studies, genomic sequencing is beginning to be covered by some insurance companies. These studies also suggest that clinical teams are in an ideal position to decide which babies should be offered these tests. However, for some cases, other barriers might limit access to these tests in the NICU.

Barriers to the implementation of genomic sequencing in the NICU include additional educational needs of non-genetics providers, clarification of professional roles for neonatal and genetics providers, lack of efficient workflows to manage logistics, concerns regarding the complexity of the information that could be learned from the test, insurance coverage, and geographic and financial access [22,23]. This commentary aims to review barriers that may impact access to genomic sequencing in the NICU, discuss the ethical implications of potential barriers to genomic testing, explore ways in which access to testing might be enhanced, and offer suggestions for future research in these areas.

## 2. Factors Impacting the Variability in Access to Genomic Sequencing

### 2.1. Insurance Approval Requirements

Currently in United States NICUs, genetic testing may be paid for by any one or a combination of the following parties: hospital, neonate’s family, private funding, community funding, insurance payer, and/or government sources. In an inpatient setting, this testing often goes through institutional billing, where the bill is generated by the institution and becomes part of daily NICU charges. Costs that are not covered by the payer are paid through a combination of the hospital and patient. The variability in these costs is largely moderated by hospital policy regarding balance billing, the patient’s insurance plan, and demonstrated financial abilities of the patient. One common criticism of implementing genomic testing as part of a newborn screening program is the high cost. These are valuable concerns, but the cost of genomic testing has continued to decline since its implementation into clinical care and may be cost-effective in managing infants in high acuity NICU settings [8,9]. Currently, since genomic technologies are not typically embedded into federal or state programs, each hospital manages their financial risk related to implementation of this testing in different ways. Many hospitals have checks and balances in place to protect financial loss, such as committee review of medical necessity or prior-authorization requirements for genetic testing. Prior reports have noted prior-authorization to be a pressing barrier to implementing WES, highlighting the tension between widespread implementation and equitable access [24]. Therein lies a tension between financial protection of an organization at the cost of providing equitable care for all neonates in this setting.

In some NICUs, insurance approval is required prior to initiating genetic testing. Even for patients with similar phenotypes, insurance coverage may differ based on the payer, as reimbursement policies for genetic testing are not well-established [23]. In one study of over 1000 people of varying races/ethnicities, blacks were significantly more likely to be insured through Medicaid/Medicare than non-Hispanic whites, which may decrease access to genetic testing if dependent upon insurance approval [25]. In our experience, public payers, such as Medicaid, typically lag behind private insurers in providing approval for medically indicated genetic testing. In this way, instituting insurance approval requirements during an inpatient stay might increase health disparities. Although genome sequencing in high acuity NICUs has been demonstrated to provide more diagnoses, and possibly improve clinical outcomes, insurance denials remain common [19].

**Figure 1:**
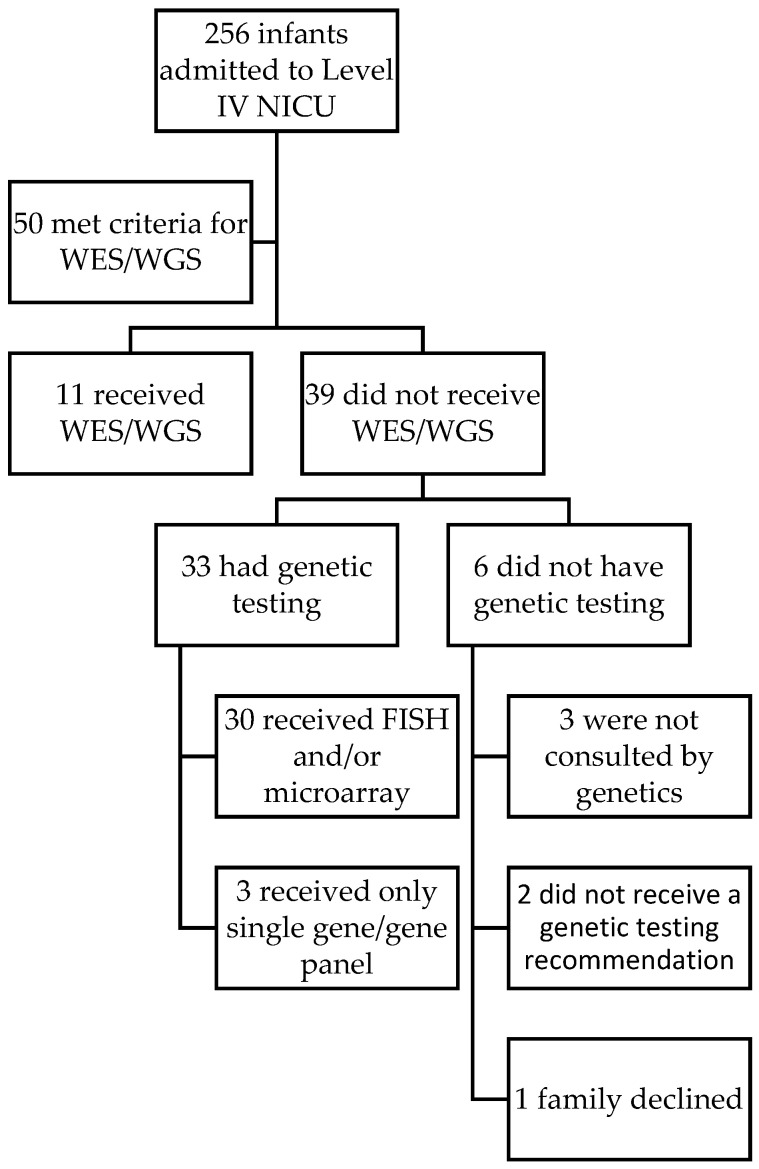
Uptake of WES/WGS. 50 neonates met criteria for WES/WGS. However, only 22% (11/50) received it within the study timeframe. Of the 39 who did not receive WES/WGS, only 1 family declined genetic testing. Of the remaining 39, six received no genetic testing and 33 received other genetic testing that did not include WES/WGS.

To further explore the potential relevance of these considerations, we reviewed 256 infants <120 days old admitted to a single Level IV NICU during a time in which the hospital required insurance approval prior to completing inpatient genetic testing. IRB approval was obtained for this review. During a 6-month period in 2017, 50 infants were eligible for genomic sequencing based on specific criteria [26]. These criteria were similar to those commonly used in a NICU setting for determining clinical trial inclusion for rapid WES and/or WGS [11,12,14,15,16,26]. At that time, few insurance companies were approving clinical WES for inpatients and no insurance company was approving clinical WGS [27]. Most infants (45/50) received genetics consults in the NICU. However, only 22% (11/50) received WES/WGS. Of the 39 neonates who did not, 30 received FISH and/or chromosomal microarray (CMA) with or without single gene or gene panel testing, 3 received only a single gene or gene panel testing, and 6 did not receive any genetic. Among those who did not get tested, one family declined testing, and two cases who were consulted by genetics did not receive any testing recommendation (Figure 1). Is it possible that the need for insurance pre-approval played a role in explaining why there were relatively few patients who received WES/WGS? Regardless of the answer, should genetic testing for acutely ill infants be limited by insurance pre-approval? Does this approach ensure equitable access to a rapid genetic diagnosis?

**Figure 2:**
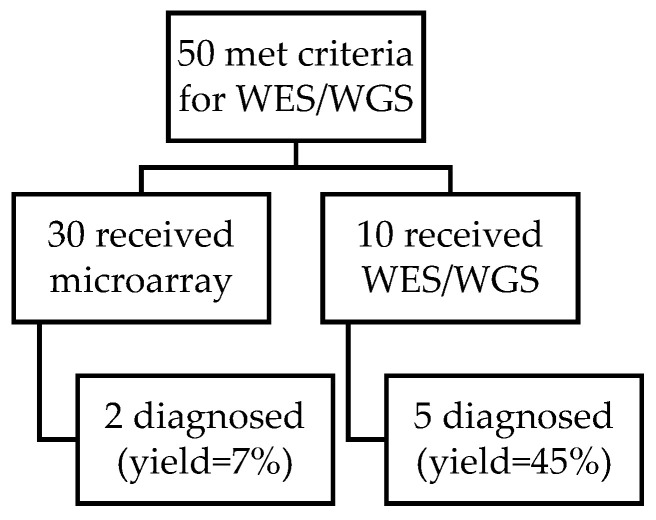
Although more neonates had FISH and/or microarray testing than WES/WGS in our cohort, the yield of microarray was much lower. The yield of microarray was 7% and the yield of WES/WGS was 45%.

Of the infants who received WES/WGS, five were diagnosed, resulting in a 45% diagnostic yield. FISH and/or CMA were used in 60% (30/50) of neonates and diagnosed 2, resulting in a 7% (2/30) diagnostic yield (Figure 2). Thus, although the utilization of WES/WGS was low, it accounted for most of the genetic diagnoses. 

**Figure 3:**
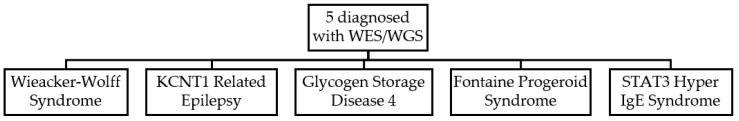
None of the conditions that were diagnosed by WES/WGS are on the RUSP.

None of the diagnoses that were made are currently on the recommended uniform screening panel (RUSP) and would have been missed by NBS alone (Figure 3). 

### 2.2. Hospital Review/Approval Committees

For hospitals where insurance approval requirements may not exist, some have developed internal review/approval committees. These committees may be led by local genetics specialists/other experts to evaluate which inpatients may be best suited to receive genomic sequencing based on certain criteria. Common criteria include assessment of phenotype and likelihood of changes to clinical management. These committees are designed to facilitate genomic sequencing by approving testing for the sickest infants at the institutional level, curbing insurance approval and minimizing financial loss. With this model, infants who are most likely to receive testing are those that are perceived by the local committees and/or particular individuals as having the highest likelihood of having a genetic condition and/or changing medical management. Committee approval may allow for testing beyond what the neonate’s payer would cover compared to going through insurance alone. However, other neonates with similarly complex symptoms that are less well understood may not be granted similar access to testing if a change of management is less likely or perceived as less necessary, based on current knowledge about these conditions. It is known that requiring a suspicion of a genetic disorder as their reason for admission will miss about half of patients with a genetic etiology [6,18,23]. Given the vast evolution of our knowledge and technology in detecting rare genetic conditions, should we limit testing to those with a subjectively “higher risk” than other neonates judged by local experts to be at “lower risk” for genetic disease? Are we facilitating equity for infants who may also have an underlying genetic etiology, but whose phenotype has not been studied as extensively or is not as recognizable?

### 2.3. Inconsistent Definitions of “Change of Management” Impacts Insurance Coverage Policies

What is defined by “change of management” is not standardized across clinical trials/studies exploring the impact of WES/WGS. Previously published definitions of this term have included recommendations for additional testing, specialty consultation, specific medical and/or surgical treatment, change in recurrence counseling, transfer of care, and/or redirection of care [10,13,14]. However, these outcomes may not align with insurance payers’ perspectives on what would be an “acceptable” change of management to warrant insurance coverage. A study by Trosman et al. surveyed and interviewed executives from 14 different insurance payers to learn more about their perspectives on insurance coverage for exome sequencing [28]. Most participants agreed that the impact on patient care/clinical management was most important when considering coverage. However, all participants agreed that reproductive risk information or knowledge that could impact diagnosis in other family members was not enough for coverage on its own. Additional discussion between clinical and payer stakeholders is warranted to determine whether including reproductive/genetic counseling outcomes as a “change of management” might be appropriate. Harmonizing what outcomes should be defined by the term “change of management” between research studies and insurance payers is necessary so that the available evidence regarding this variable aligns with what is most likely to provide evidence of medical necessity. Additionally, payers ought to be more transparent about what evidence/criteria they use to make approval decisions about next-generation sequencing and what pieces of evidence are most likely to influence claim decisions. This will allow for meta-analysis and meaningful appeals to alter payer denial patterns and/or revisions to insurance policy. 

## 3. Ethical Implications for Barriers to Genomic Sequencing

### 3.1. Legal Implications

Underlying newborn medical care (and healthcare overall) is a moral obligation to protect children, especially vulnerable newborns, who are unable to protect themselves, regardless of economic considerations. In many countries, this extends to larger legal obligations. Some NICUs are nested within children’s hospitals that are tax exempt, non-profit organizations. US law of tax-exempt entities suggests that these organizations need to provide care regardless of the ability of families to pay and/or of insurance coverage. 

Barriers such as requirements for insurance prior-authorization have been shown to alter physician prescribing patterns towards being more conservative [29]. Therefore, it is possible that a geneticist’s desire to recommend testing may be confounded by their prior awareness/experience of being unable to obtain coverage for WES/WGS with the neonate’s payer, especially if prior peer-to-peer and/or appeals have not altered denial decisions for similarly complex situations in the past. Under-recommendation of genomic sequencing due to prior experience in navigating payer decisions may lead to genomic malpractice suits. These suits may be pursued by neonates’ families in situations where a genetic test with known clinical utility was not offered by the clinical team. This genetic information may have clarified recurrence risk. Wrongful conception was upheld in court for a family who had two children with Fragile X, when they were not made aware that their first child had this condition (“Molloy v. Meier,” 2003). Thus, the absence of a genetic diagnosis may potentially lead to future wrongful conception claims for physicians who did not recommend medically indicated testing. It has also been postulated that insurers may be liable if a clinically appropriate test recommended by the clinical team was denied by the insurer [30]. This highlights the importance of insurance companies staying abreast of current literature regarding the clinical utility of genomic testing.

### 3.2. Psychosocial Implications for Parents and Providers

Inequitable access to genomic sequencing impacts not only the provision of medical services, but can also lead to parental and provider moral distress. Parents of children with undiagnosed diseases are known to experience emotions such as anxiety, depression, uncertainty, anger, powerlessness, frustration, and denial [31,32,33,34]. One study identified this prevalence to be 40%, with parents with older children and longer duration of illness having lower depressive and anxious symptoms and better coping self-efficacy [33]. An additional qualitative study interviewing parents of children with undiagnosed disease regarding coping with the diagnostic processes identified that it was an important piece of the coping process to know that everything possible had been done for their child. They also discussed a personal responsibility to follow-up with doctors, read the medical literature, ask for second opinions, and seek alternative treatment options. This was motivated by a fear for the possibility of realizing in the future that they failed their child if they did not access something that could have helped them [31]. Thus, unequitable distribution of genomic tests may contribute further to this fear and facilitate challenges to parental coping throughout the diagnostic process. 

From the parental perspective, waiting for a medically indicated test is not common for other tests ordered in the NICU. For example, imaging and surgery can be done without insurance or hospital approval. These interventions cost thousands of healthcare dollars, may at times have similarly uncertain outcomes, and may have clinical utility that is not well-understood. Thus, when genetics specialists believe that a genetic test is needed for diagnostic purposes with hopes to end the diagnostic odyssey, it is likely distressing for both medical providers and families to wait, albeit genetic tests are much cheaper than other medical interventions that are done routinely without insurance approval. For most parents who participated in a qualitative study, when their expectations about their child’s care were not met, such as finding a diagnosis or accessing diagnostic tools/treatment, they experienced serious frustration and distrust, increasing their feeling of powerlessness [31]. In addition to considering the impact of not having a diagnosis on families’ coping, these emotions can also impact the patient-provider relationship. 

Moral distress has been noted to have a higher prevalence in palliative, emergency, intensive, and pediatric providers. In one study, the leading causes of moral distress included lack of resources, lack of administrative action, providing false hope, and excessive documentation that interferes with patient care [35]. More recently, it has also been reported amongst genetics specialists who see patients in an inpatient setting [36,37,38]. Specifically, one recent qualitative study found that a common source of moral distress for genetics providers was related to lack of/poor insurance coverage for clinically indicated genetic testing for their patients [37]. In our experience, it is distressing to providers who have recommended a genetic test to not be able to utilize the results as part of inpatient medical management and counseling, when suspicion for a genetic etiology is high. A prior study of NICU nurses identified that moral distress related to the hospital ethical climate was a significant factor for them to consider leaving their institution [39]. This finding is concerning for genetics providers who already report high levels of distress and burnout and see a reduction in medical trainees pursuing the field. However, this phenomenon has not been studied for genetics professionals in the NICU as well as whether the presence of barriers to genetic testing contributes to the ‘hospital ethical climate’ or moral distress, 

## 4. Considerations to Promote Access to Genomic Sequencing

### 4.1. Standardized Approval of Genomic Sequencing for All Neonates with Phenotype Known to Have High Yield

One approach to facilitate access to genomic testing on a population-based scale for infants in a Level IV NICU is to consider the standardized approval of genomic sequencing for neonates who are known to have the highest chance of benefit, regardless of financial status and/or insurance policy. Providing WES as a first-tier test for all infants meeting similar inclusion/exclusion criteria may prove beneficial, as has recently been suggested for individuals with neurodevelopmental delays [40]. Currently, data has shown patients with seizures and/or neurodevelopmental phenotypes to have a high yield with WES, affecting management 41–48% of the time [24,41]. Additionally, patients with inborn errors of metabolism have been reported to have yields as high as 68% with WES technology [42]. Another study identified at least a 25% yield for patients who received WES with phenotypes associated with muscular dystrophies/myopathies, dermatologic conditions, multiple congenital anomalies, skeletal dysplasias, hypotonia, cardiac disease, metabolic disorders, hematologic conditions, seizures, and others [43]. These presentations are common in a Level IV NICU setting. Providing access to genomic testing for neonates with these characteristics, regardless of their financial and/or insurance status, would promote equity [8]. Even broader approaches, such as offering genomic sequencing for all neonates in a Level IV NICU may identify additional infants with conditions that are relatively rare but may alter medical management for that child or other children in the future. 

### 4.2. Creative Technological Solutions to Extend Genetic Evaluation to Areas without Genetics Experts 

Genetic disease is common in infants admitted to any NICU and is likely enriched in Level IV NICU’s. However, Level III NICUs admit infants with genetic disease including aneuploidy, sexual differences of development, encephalopathy, hypotonia, hyperinsulinism, milder forms of known genetic conditions that we may not currently understand, and/or rarer and milder genetic conditions not requiring admission to a Level IV NICU. Thus, for any NICU without direct access to genetics expertise, it would be reasonable to pursue creative technological solutions to enable genetic evaluation. There are opportunities for mobile application development, interactive education modules for neonatal trainees and providers, and decision support tools for neonatal providers. These efforts ought to occur in collaboration with genetics experts including board-certified geneticists and genetic counselors.

## 5. Opportunities for Future Research

- Measure the short- and long-term healthcare outcomes for critically ill neonates, stratified by phenotype, who did and did not receive genomic sequencing while in the NICU.
- Characterize social indicators that may predict health disparities regarding access to genomic sequencing.
- Evaluate the criteria used to make decisions about which patients can access genomic sequencing within hospital-based committee structures.
- Explore whether prior-authorization and/or hospital-based committee approval for genomic sequencing in the inpatient setting contributes to the perceived ‘hospital ethical climate’ and/or moral distress for genetics specialists and other neonatal providers.
- Explore the parental experience of navigating prior authorization, hospital-based committee approval, and/or other barriers to genomic sequencing in the NICU to better understand their psychosocial impact. Although prior research identified psychosocial distress in parents of undiagnosed children, few studies have occurred in parents of newborns who are still in the hospital.
- Development and implementation of mobile applications and/or decision support tools for neonatal providers to extend genetics evaluation to NICUs in geographic areas where this access does not exist or is limited.
- Development and implementation of interactive education modules for neonatal trainees and/or current neonatal providers to support their decision making, triage, and advocacy for access to genomic sequencing for their patients.

## 6. Conclusions

Newborn screening is a successful public health program implemented in numerous countries across the world for the early identification of infants with rare genetic disease to prevent morbidity and mortality. However, in the United States, neonates in a NICU are unlikely to receive optimal benefit from this screen compared to healthy infants. Thus, neonates admitted to a NICU setting would likely benefit from wider screening, to include consideration of genomic sequencing. In lieu of this testing being offered as part of federal or state efforts, we suspect that several barriers and social forces impact access to genetic testing in NICUs of varying sizes and levels across the United States. Our review considers the potential barriers that might stand in the way of equitable assessment of vulnerable infants in a setting that is known to exacerbate health disparities. There may be opportunities for hospitals to lower risks and cost while fostering beneficence for neonates by routinely facilitating genomic testing for infants with certain phenotypes with high yield such as neurodevelopmental conditions including seizures, muscular dystrophies/myopathies, dermatologic conditions, multiple congenital anomalies, skeletal dysplasias, hypotonia, cardiac disease, metabolic disorders, and hematologic conditions. Further, this may suggest an opportunity for payers to partner with hospitals and NICU/genetics experts to foster standardized approval practices for infants with complex healthcare needs. The tension between financial risks and beneficence must be addressed before considering the widespread implementation of genomic testing in this population to promote equitable care for the most vulnerable and acutely ill infants in the United States and across the globe.

## Data Availability

The data presented in this study are available within the text of this article.
